# Tobacco Knowledge among Adults in Zhejiang Province, China

**DOI:** 10.1371/journal.pone.0059172

**Published:** 2013-03-20

**Authors:** Yue Xu, ShuiYang Xu, QingQing Wu, YuJie Guo

**Affiliations:** Zhejiang Center for Disease Control and Prevention, Hangzhou, China; University of New South Wales, Australia

## Abstract

**Objective:**

The aims of current study were to assess the level of tobacco knowledge, anti-tobacco messages and major information channels in Zhejiang.

**Methods:**

Face-to-face interviews were conducted with 2112 adults in Zhejiang. Data on demographic information, smoking status, tobacco knowledge, anti-tobacco messages and major information channels was collected.

**Results:**

The findings revealed that only 31.87% of the population were aware that smoking could cause all three diseases (stoke, heart disease, and lung cancer), 86.09% were aware that smoking causes lung cancer, 46.43% and 42.40% were aware that smoking causes stroke and heart attack, respectively. Residence and education level had significant effects on awareness, while the effects of smoking status, gender, age, and household monthly income were not significant. There were five major information channels as follows: television (67.52%), newspapers or magazines (40.79%), billboards (30.02%), public walls (24.72), and radio (23.79%). Respondents got the following anti-tobacco messages from mass media: “No smoking in public” (66.34%), “No smoking in front of other people” (35.18%) and “Not offering cigarettes to one another” (22.82%).

**Conclusions:**

The tobacco knowledge among residents in Zhejiang province is relatively poor. Improved information channels and content of anti-tobacco messages are necessary to increase the public’s tobacco knowledge, particularly among rural residents and people with less education.

## Introduction

Tobacco use is a major cause of preventable disease and premature death. The tobacco epidemic is responsible for 5.4 million deaths annually and killed 100 million people worldwide in the last century [1]. Though tobacco use is steadily declining in developed countries, smoking prevalence and cigarette consumption are increasing in many developing countries. China is the world’s largest consumer of tobacco products, with an estimated 301 million smokers [2], and the annual number of deaths caused by tobacco use now exceeds 1 million. If the current trends are maintained, that number will rise to over 2 million by 2030 and to 3 million by 2050 [3]. The World Health Organization (WHO) has provided global leadership to promote its Framework Convention on Tobacco Control (FCTC) [4], in a bid to halt the worldwide tobacco epidemic. Implementation of the FCTC started in China in 2006.

Zhejiang is one of the smallest province-level political units of China, but it is also one of the most densely populated and affluent. In the last 4 years, the local government authorities have paid certain attention to tobacco control practice, which was well accepted by local society. In the provincial capital, Hangzhou municipality expanded a smoking ban to hospitals, kindergartens, schools, libraries and stadiums in 2010. Since 2003, many intervention campaigns about tobacco control issues were conducted, to increase public awareness of tobacco knowledge. Local CDC had launched a healthy school program with tobacco use prevention in 4 schools [5] in 2003, smoke-free intervention in 20 health institutions [6] in 2009, and promotion campaigns to mark World No Tobacco Day every year, which focused on educating smokers about the risks of smoking. Each of these campaigns provided tobacco knowledge to the public, and likely laid the groundwork for the denormalization of smoking.

Studies in China and elsewhere indicate that improved tobacco knowledge can reduce tobacco consumption among adult smokers, and enhance smokers’ intention to quit smoking [7]–[8]. Therefore, it is urgently needed to raise levels of tobacco knowledge. Devising methods for effective monitoring of the tobacco knowledge and obtaining representative data are crucial steps toward this goal. The current study sought to obtain scientific evidence to aid the development of tobacco control and health education strategies in the future, through an in-depth analysis of data from the Tobacco Control China baseline survey regarding tobacco knowledge.

## Methods

### Participants

The target population for the survey was defined as all residents, aged 18 years and older, living in Zhejiang province, excluding those living in student dormitories, military barracks, prisons, or hospitals.

### Sampling Design

**Figure 1:**
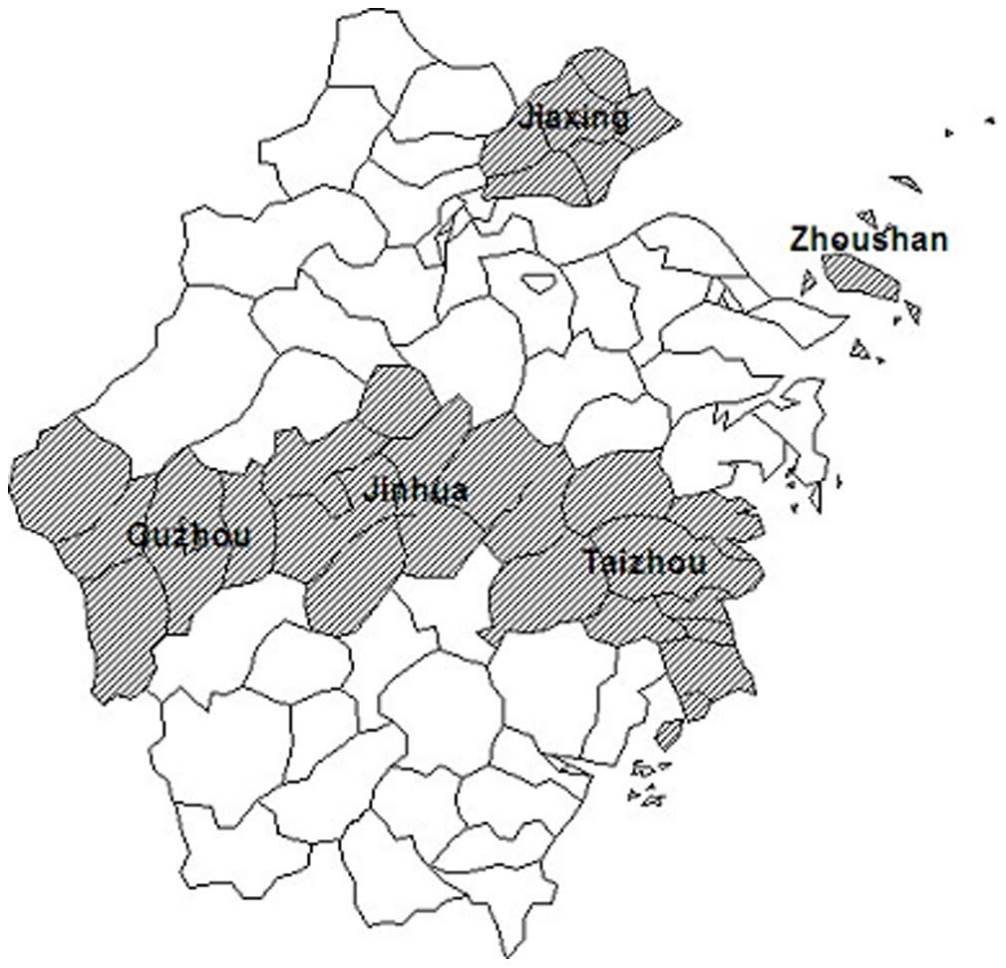
The geographical distribution of the 5 regions in Zhejiang.

A multi-stage stratified cluster sampling design was implemented in the survey. The 5 regions were selected based on their geographic locations (see fig. 1). In the first stage, each region was further divided into urban and rural areas, making 10 strata in total. In the second stage, each stratum was partitioned into several segments of around 50 households (using mapping and listing to determine the number). In the third stage, 6 segments were randomly selected from each stratum, and every household in the selected segment was visited. Finally, one eligible household member 18 years and older from each participating household was randomly sampled for an interview.

### Data Collection

The Epidemiology and Intervention Research for Tobacco Control in China is a tobacco control intervention study at the provincial level. The baseline survey was conducted between May and October 2010. All the survey interviewers and supervisors were trained by Peking Union Medical College staff. The training sessions took place in small groups and were given by the same trainers to ensure consistency. Before the interview, mapping and listing was conducted by local CDC staff to identify each selected household. The survey was conducted in Mandarin through face to face interviews with informed consent obtained from the respondents. Data on demographic information, smoking status, tobacco knowledge, anti-tobacco messages and major information channels was collected. Survey information was collected using paper. Up to three visits to a household were made to interview the target person within that household. A total of 2112 interviews were completed, and the household response rate was 92.31%.

### Measures

#### Tobacco knowledge

Tobacco knowledge is the dependent variable used in this analysis and was measured by asking if respondents agreed with the following statements: 1) “smoking causes heart disease”; 2) “smoking causes lung cancer”; and 3) “smoking causes stroke”. Response categories were “yes” and “no”. Respondents were classified as having tobacco knowledge for each of the statements with which they agreed.

The following independent variables included in the analysis were obtained through self-report: residence (urban, rural); gender (male, female); age (18–34 years, 35–44 years, 45–54 years, 55 years or older); education level (primary school complete or less, secondary school complete, high school complete, college complete or above); household monthly income (low: <1000 *Yuan*, medium: 1000 to 4999 *Yuan*, high:≥5000 *Yuan*) and smoking status (smoker, non-smoker). Respondents who were smoking at the time of the survey were classified as smokers. Respondents who were not smoking at the time of the survey, including former smokers and never smokers, were classified as non-smokers.

### Message Channels and Exposure to Specific Anti-tobacco Messages

Anti-tobacco message channels were assessed by asking: “During the past 30 days, have you noticed anti-tobacco messages in the following channels?”: 1) in newspapers or in magazines; 2) on television; 3) on billboards; 4) on posters or promotional materials; 5) on public walls; 6) in cinemas; 7) on the Internet; 8) on public transportation vehicles; and 9) other places. Response options included “yes” and “no”. Exposure to specific anti-tobacco messages was measured by asking if respondents got the following information from mass media in the last 1 year: 1) “No smoking in public”; 2) “No smoking in front of other people”; and 3) “Not offering cigarettes to one another”. Response categories were “yes” and “no”.

### Statistical Analysis

SPSS version 18.0 was used for all analyses. Descriptive statistics were conducted on the demographic variables, smoking status, and other parameters which might be associated with tobacco knowledge. Survey logistic regression was applied to determine factors associated with tobacco knowledge.

### Ethics

This study was conducted with the approval of the Institute of Basic Medical Sciences of Chinese Academy of Medical Sciences, and the Internal Review Boards at Zhejiang Center for Disease Control and Prevention (Hangzhou, China). The survey was conducted through face to face interviews with written informed consent obtained from the respondents and their guardians. To protect the participants’ confidentiality, we kept all data confidential and without identifiers.

## Results

### General Information

The study was conducted in 10 counties/county-level cities. Valid interviews were conducted with 2112 respondents, of whom 1050 were male and 1062 were female. There were 1070 urban residents and 1042 rural residents among the respondents, representing a population which most commonly had completed primary school or less (36.13%), followed by completion of secondary school (34.19%), high school (16.43%), and college or more education (13.21%). Regarding income level, 34.85% reported household monthly income less than 1000 *Yuan*, 58.10% reported between 1000 to 4999 *Yuan*, and 6.53% reported more than 5000 *Yuan*.

### Tobacco Knowledge

Table 1 shows level of tobacco knowledge by various socio-demographic factors. Only 31.87% of Zhejiang adult residents knew that smoking could cause all three diseases (stroke, heart disease, and lung cancer), 86.09% were aware that smoking causes lung cancer, 46.43% and 42.40% were aware that smoking causes stroke and heart attack, respectively. The percentage of awareness of tobacco knowledge varied among different subgroups of residence, age, education and income level. It was lower among rural residents than their urban counterparts. Older respondents tended to have lower awareness levels. Among the oldest age group (55 years or older), just one quarter knew that smoking is a cause of three diseases: stroke, heart disease, and lung cancer. Within the youngest age group (18–34 years), only 39.53% had this level of tobacco knowledge. Education level was positively associated with tobacco knowledge. Even among respondents with a college education lor more, only 63.41% were aware that smoking is a cause of all three diseases. The rate of awareness was only 22.54% among those who completed their primary education or less. Compared with respondent with middle and high incomes, respondents with low income had a lower level of tobacco knowledge (22.96%).

**Table 1:** Percentage of respondents with knowledge of tobacco-related disease.

Socio-demographic	Adults who believe that smoking causes the diseases (Percentage of survey respondents)			
	Stroke	Heart attack	Lung cancer	All three diseases
**Smoking status**				
Smoker	220 (39.43)	240 (43.01)	482 (85.77)	172 (29.05)
Non-smoker	566 (38.82)	614 (42.17)	1269 (86.21)	501 (32.96)
**Residence**				
Urban	498 (48.68)	505 (49.41)	917 (88.34)	396 (37.01)
Rural	288 (29.00)	349 (35.18)	834 (83.73)	277 (26.58)
**Age**				
18–34 years	80 (47.62)	81 (48.21)	154 (91.67)	68 (39.53)
35–44 years	318 (40.25)	354 (44.81)	726 (90.98)	297 (36.18)
45–54 years	345 (37.34)	375 (40.63)	770 (82.53)	273 (27.89)
55 years or older	43 (32.09)	44 (33.08)	101 (74.81)	35 (25.00)
**Education level**				
Primary school complete or less	222 (30.71)	234 (32.41)	546 (75.00)	172 (22.54)
Secondary school complete	260 (37.85)	284 (41.34)	619 (88.94)	214 (29.64)
High school complete	149 (44.88)	160 (48.34)	322 (96.12)	138 (39.77)
College complete or above	155 (56.78)	175 (64.10)	263 (95.99)	149 (63.41)
**Household monthly income**				
Low	210 (29.83)	235 (33.43)	566 (79.94)	169 (22.96)
Medium	521 (44.61)	559 (47.90)	1072 (90.69)	449 (36.59)
High	48 (36.09)	51 (38.38)	103 (77.44)	47 (34.06)
**Overall**	786 (46.43)	854 (42.40)	1751 (86.09)	673 (31.87)

### Factors Associated with Tobacco Knowledge

A multivariate complex sampling logistic analysis was conducted on six factors (smoking status, residence, gender, age, education level, and household monthly income). Of the factors listed above, residence and education level had significant effects on knowledge, while the effects of smoking status, gender, age, and household monthly income were not significant. Knowledge levels were significantly higher among urban residents compared with rural residents, and among those with a higher education level compared with those with a lower level (see Table 2).

**Table 2:** Factors associated with tobacco knowledge.

Covariate	Percentage	OR	95%CI	P Value
**Smoking status**				
Smoker	29.05	0.83	0.67 to 1.04	0.10
Non-smoker	32.96		Reference	
**Residence**				
Urban	37.01	1.22	1.00 to 1.50	0.05
Rural	26.58		Reference	
**Age**				
18–34 years	39.53	0.95	0.56 to 1.62	0.85
35–44 years	36.18	0.87	0.56 to 1.37	0.55
45–54 years	27.89	0.81	0.62 to 1.45	0.95
55 years or older	25.00		Reference	
**Education level**				
Primary school complete or less	22.54	0.32	0.22 to 0.45	0.00
Secondary school complete	29.64	0.41	0.30 to 0.56	0.00
High school complete	39.77	0.61	0.44 to 0.85	0.00
College complete or above	63.41		Reference	
**Household monthly income**				
Low	169 (22.96)	0.75	0.50 to 1.14	0.18
Medium	449 (36.59)	1.15	0.78 to 1.69	0.48
High	47 (34.06)		Reference	

### Message Channels

There were five major channels through which the public had exposure to anti-tobacco messages, including television (67.23%), newspapers/magazines (40.48%), billboards (29.73%), public walls (24.43%), and radio (23.53%). Rural residents had less exposure to anti-tobacco messages than their urban counterparts through every major message channel (see details in Table 3). Among respondents age 18–34 years, 36.63% were exposed to anti-tobacco messages through public transportation vehicles, and 32.56% through the Internet. The education distribution showed a step gradient; respondents with higher education were substantively exposed to anti-tobacco messages through a greater number of channels than their counterparts with less education. Compared to those in the lowest income level, respondents with middle and high income had greater exposure to anti-tobacco messages.

**Table 3:** Channels through which respondents got the anti-tobacco messages (past 30 days).

Socio-demographic	Number of survey respondents who indicated that they had obtained information from this channel in the last thirty days (Percentage of survey respondents)								
	Newspapers/magazines	Television	Radio	Billboards	Posters/promotion materials	Public walls	Cinemas	Internet	Public transportation vehicles
**Residence**									
Urban	479 (44.77)	762 (71.21)	279 (26.07)	358 (22.99)	246 (23.36)	301 (28.13)	128 (11.96)	178 (16.64)	279 (26.07)
Rural	376 (36.08)	658 (63.15)	218 (20.92)	270 (25.91)	125 (12.00)	215 (20.63)	65 (6.24)	80 (7.68)	198 (19.00)
**Age**									
18–34 years	94 (54.65)	127 (73.84)	48 (27.91)	76 (44.19)	41 (23.84)	63 (36.63)	32 (18.60)	56 (32.56)	63 (36.63)
35–44 years	362 (44.09)	574 (69.91)	213 (25.94)	271 (33.01)	160 (19.49)	245 (29.84)	95 (11.57)	138 (16.81)	220 (26.80)
45–54 years	360 (36.77)	637 (65.07)	210 (21.45)	255 (26.05)	159 (16.24)	190 (19.41)	65 (6.64)	62 (6.33)	178 (18.18)
55 years or older	39 (27.86)	82 (58.57)	26 (18.57)	26 (18.57)	11 (7.86)	18 (12.86)	1 (0.71)	2 (1.43)	16 (11.43)
**Education level**									
Primary school complete or less	205 (26.87)	445 (58.32)	146 (19.13)	134 (17.56)	102 (13.37)	102 (13.37)	28 (3.67)	26 (3.41)	86 (11.27)
Secondary school complete	306 (42.38)	520 (72.02)	182 (25.21)	233 (32.27)	113 (15.65)	177 (24.52)	51 (7.06)	67 (9.28)	170 (23.55)
High school complete	188 (54.18)	248 (71.47)	101 (29.11)	152 (43.80)	80 (23.05)	124 (35.73)	55 (15.85)	77 (22.19)	125 (36.02)
College complete or above	155 (55.56)	206 (73.84)	67 (24.01)	108 (37.71)	76 (27.24)	113 (40.50)	59 (21.15)	88 (31.54)	96 (34.41)
**Household monthly income**									
Low	222 (30.16)	442 (60.05)	139 (18.89)	168 (22.83)	89 (12.09)	139 (18.89)	47 (6.39)	59 (8.02)	136 (18.48)
Medium	558 (45.48)	877 (71.89)	317 (25.84)	412 (33.58)	247 (20.13)	343 (27.95)	130 (10.59)	172 (14. 02)	302 (24.61)
High	66 (47.83)	90 (65.22)	37 (26.81)	46 (33.33)	33 (23.91)	33 (23.91)	15 (10.87)	26 (18. 84)	38 (27.54)
**Overall**	855 (40.48)	1420 (67.23)	497 (23.53)	628 (29.73)	371 (17.57)	516 (24.43)	193 (9.14)	258 (12.22)	477 (22.59)

Table 4 shows the prevalence of respondents who got anti-tobacco messages from mass media by various socio-demographic factors. 66.34% of respondents got the anti-tobacco message “No smoking in public”, though only 35.18% and 22.82% got the message “No smoking in front of other people” and “Not offering cigarettes to one another”, respectively. The level of anti-tobacco knowledge varied considerably by residence, age, the level of education and household monthly income.

**Table 4:** Anti-tobacco messages which respondents got from mass media (past year).

Socio-demographic	Number of survey respondents indicating they had received the following anti-smoking messages (Percentage of survey respondents)		
	No smoking in public	No smoking in frontof other people	Not offering cigarettesto one another
**Residence**			
Urban	763 (71.31)	399 (37.29)	226 (21.12)
Rural	638 (61.23)	344 (33.01)	256 (24.57)
**Age**			
18–34 years	125 (72.67)	64 (37.21)	40 (23.26)
35–44 years	594 (72.35)	331 (40.32)	202 (24.60)
45–54 years	608 (62.10)	305 (31.15)	211 (21.55)
55 years or older	74 (52.86)	43 (30.71)	29 (20.71)
**Education level**			
Primary school complete or less	390 (51.11)	199 (26.08)	128 (16.78)
Secondary school complete	532 (73.68)	284 (39.34)	194 (26.87)
High school complete	245 (70.61)	129 (37.18)	92 (26.51)
College complete or above	234 (83.87)	131 (46.95)	68 (24.37)
**Household monthly income**			
Low	424 (57.61)	218 (29.62)	146 (19.84)
Medium	877 (71.48)	465 (37.90)	294 (23.96)
High	91 (65.94)	52 (37. 68)	35 (25.36)
**Overall**	1401 (66.34)	743 (35.18)	482 (22.82)

## Discussion

The study, to our knowledge, is the first one to assess the level of tobacco knowledge, major anti-tobacco message channels and exposure to specific anti-tobacco messages in Zhejiang, one of most densely populated provinces of China (463.7/km^2^) [9]. The major findings of the current study include: (1) less than one third of the residents of Zhejiang know that smoking is a cause of all three of the diseases measured (stroke, heart disease, and lung cancer); many respondents don’t have specific tobacco knowledge; (2) rural residents and those with less education have less tobacco knowledge than urban residents and those with greater education, and may therefore benefit from health education and tobacco control efforts; (3) anti-tobacco messages were disseminated through five major channels (television, newspapers/magazines, billboards, public walls, and radio); public transportation vehicles and the Internet were important information channels for young people; (4) exposure to anti-tobacco messages in mass media was relatively poor.

From the results of this survey, we found that about 31.87% of the adults in Zhejiang knew that smoking causes all three of the following diseases: stroke, heart disease, and lung cancer. Compared to the results of the 2010 Global Adult Tobacco Survey in China (23.2%) [10], the level of tobacco knowledge was higher than the national average, but still far below the levels of developed countries [11]–[12] (90.8% in Canada and 88.7% in Australia). Studies show that increases in health knowledge are strongly associated with reductions in smoking initiation, increases in cessation behaviour and long-term smoking abstinence [1], [13]–[14]. The current low level of tobacco knowledge in Zhejiang suggests that China may observe a marked reduction in smoking rates in response to tobacco control efforts. We should engage in campaigns to educate the public on tobacco knowledge. Use of graphic health warnings on tobacco packaging have been effective in other countries and could be an effective means to communicate specific disease risks in China [15].

More than 80% of respondents agreed that smoking could cause lung cancer. However, the findings reveal major gaps in the knowledge of other health effects. For example, less than 50% of respondents endorsed smoking as a risk factor for stroke and heart attack, respectively- both leading causes of death in China [16]. This phenomenon is possibly due to the strong emphasis health education campaigns have placed on this fact in China. The depth and breadth of tobacco knowledge information promoted to the public are limited, and may therefore have little effect in terms of changing people’s tobacco use behaviour [7], [11], [17]. More adequate tobacco health education strategies should be applied to disseminate other harmful consequences of smoking, such as heart disease and stroke.

In order to improve strategies for communicating information about tobacco knowledge, this paper analyzes the socio-demographics of tobacco knowledge among adults. We found that awareness regarding tobacco knowledge across all participants was significantly associated with residence and education levels. Rural residents and people with less education have less tobacco knowledge than urban residents and those with greater education, and may therefore benefit from health education and tobacco control efforts were the focus groups for health education on tobacco control, it should be strengthened to improve the awareness of tobacco knowledge of the whole population. In addition, we also found some differences in tobacco knowledge among adults. First, an important finding in this study is the high awareness regarding tobacco knowledge in the young people (18–34 years). The reason might be young people got more rounded education because of economic development in China. Second, there were no statistically significant associations between tobacco knowledge and income levels or smoking status. Previous research indicates that increased wealth is not associated with decreased tobacco use in China [18]. A possible interpretation for this might be cultural differences, which is an area for further research.

The findings also indicate that there were five major information channels in Zhejiang. Television (67.52%) and newspapers/magazines (40.48%) reach the greatest proportion of Zhejiang residents, though exposure through these channels is lower than in developed countries [12]. However, with regard to young people, public transportation vehicles and the Internet were also the major information channels. With China’s rapid economic development, young people spend more time traveling to work [19], and a lot of time on the Internet. The role of mass media in promoting and reducing tobacco use in the United States is now well-documented [20]–[22]. Therefore, we should use different media sources to promote specific anti-tobacco messages for different people. For example, we could use in-car television sets to have highlighted the risks of impotence for young.

Another interesting finding is that anti-tobacco messages in the mass media were relatively ineffective in terms of being associated with improved tobacco knowledge of the survey participants. One possible reason that about two-thirds of respondents got the message “No smoking in public,” is because the Chinese government has actively promoted the introduction of a smoke-free environment in public places in recent years [23]–[25]. However, only about one-third and one-fifth got the message “No smoking in front of other people” and “Not offering cigarettes to one another”, respectively. In China, more than two-thirds were unaware of the hazards of secondhand smoke [26], and offering cigarettes to one another is a means of social interaction and a friendly gesture [12]. This is a serious public health and social problem which may be addressed using specific anti-tobacco messages in health promotion campaigns.

A key advantage of this study is that the sample was properly selected and the sample size is large enough to be representative of the province. However, there are some limitations to this study. First, the survey used respondent self-reports to provide information, which may be subject to recall bias and social desirability. The findings were limited by potential non-response differentials. However, the weighting procedure used in the analyses may help reduce the bias. Second, tobacco knowledge was relatively difficult to measure. This study defined tobacco knowledge as knowledge of the relationship between smoking and stroke, heart attack and lung cancer. This measure underestimates the health hazards of tobacco use which potentially limited the findings.

### Conclusion

In summary, a cross sectional survey with multi-stage stratified cluster sampling was employed to study tobacco knowledge. The results of this study show that the population’s tobacco knowledge is relatively poor, Improved information channels and contents of anti-smoking messages are necessary to increase the public’s tobacco knowledge, particularly among rural residents and people with less education. The study also indicates that specific and mutli-dimensional health education should be applied in improving the public’s tobacco knowledge.
